# Migraine - a borderland disease to epilepsy: near it but not of it

**DOI:** 10.1186/s10194-024-01719-0

**Published:** 2024-01-26

**Authors:** Jakob Paungarttner, Martina Quartana, Lucrezia Patti, Barbora Sklenárová, Fatemeh Farham, Inés Hernando Jiménez, M. Gokcen Soylu, Irina Maria Vlad, Semih Tasdelen, Teresa Mateu, Oreste Marsico, Federica Reina, Viktoria Tischler, Christian Lampl, on behalf of the European Headache Federation School of Advanced Studies (EHF – SAS)

**Affiliations:** 1 Headache Medical Center Linz, Linz, Austria; 2 Department of Sciences for Health Promotion and Mother-and Childcare “G. D‘Alessandro“, University of Palermo, Palermo, Italy; 3 St. Anne’s University Hospital and Masaryk University, Brno, Czech Republic; 4 Headache Department, Iranian Center of Neurological Researchers, Neuroscience Institute, Tehran University of Medical Sciences, Tehran, Iran; 5 Department of Neurology, Severo Ochoa University Hospital, Madrid, Spain; 6 Department of Neurology, Bakırköy Prof. Dr. Mazhar Osman Mental Health and Neurological Diseases Education and Research Hospital, Istanbul, Turkey; 7 Department of Neurosciences, Iuliu Hațieganu University of Medicine and Pharmacy, Cluj-Napoca, Romania; 8 Department of Neurology, Istanbul Faculty of Medicine, Istanbul University, Istanbul, Turkey; 9 Department of Neurology, Vall d’Hebron University Hospital, Barcelona, Spain; 10 Department of Medical and Surgical Sciences, Magna Graecia University of Catanzaro, Catanzaro, Italy; 11 Regional Epilepsy Centre, Great Metropolitan “Bianchi-Melacrino-Morelli Hospitall“, Reggio Calabria, Italy; 12 NeuroTeam Life&Science, Spin-off University of Palermo, Palermo, Italy; 13 Department of Neurology and Stroke Unit, Konventhospital Barmherzige Brüder Linz, Linz, Austria; 14 Department of Neurology, Fundació Sanitària Mollet, Mollet del Vallès, Barcelona, Spain; 15 RoNeuro Institute for Neurological Research and Diagnostic, Cluj-Napoca, Romania

**Keywords:** Migraine, Epilepsy, Neurotransmitters, Genetics, Hemiplegic migraine, Migralepsy, Anti-seizure medication

## Abstract

**Background:**

Migraine and epilepsy are two paroxysmal chronic neurological disorders affecting a high number of individuals and being responsible for a high individual and socioeconomic burden. The link between these disorders has been of interest for decades and innovations concerning diagnosing and treatment enable new insights into their relationship.

**Findings:**

Although appearing to be distinct at first glance, both diseases exhibit a noteworthy comorbidity, shared pathophysiological pathways, and significant overlaps in characteristics like clinical manifestation or prophylactic treatment. This review aims to explore the intricate relationship between these two conditions, shedding light on shared pathophysiological foundations, genetic interdependencies, common and distinct clinical features, clinically overlapping syndromes, and therapeutic similarities. There are several shared pathophysiological mechanisms, like CSD, the likely underlying cause of migraine aura, or neurotransmitters, mainly Glutamate and GABA, which represent important roles in triggering migraine attacks and seizures. The genetic interrelations between the two disorders can be observed by taking a closer look at the group of familial hemiplegic migraines, which are caused by mutations in genes like *CACNA1A*, *ATP1A2*, or *SCN1A*. The intricate relationship is further underlined by the high number of shared clinical features, which can be observed over the entire course of migraine attacks and epileptic seizures. While the variety of the clinical manifestation of an epileptic seizure is naturally higher than that of a migraine attack, a distinction can indeed be difficult in some cases, e.g. in occipital lobe epilepsy. Moreover, triggering factors like sleep deprivation or alcohol consumption play an important role in both diseases. In the period after the seizure or migraine attack, symptoms like speech difficulties, tiredness, and yawning occur. While the actual attack of the disease usually lasts for a limited time, research indicates that individuals suffering from migraine and/or epilepsy are highly affected in their daily life, especially regarding cognitive and social aspects, a burden that is even worsened using antiseizure medication. This medication allows us to reveal further connections, as certain antiepileptics are proven to have beneficial effects on the frequency and severity of migraine and have been used as a preventive drug for both diseases over many years.

**Conclusion:**

Migraine and epilepsy show a high number of similarities in their mechanisms and clinical presentation. A deeper understanding of the intricate relationship will positively advance patient–oriented research and clinical work.

## Introduction

Migraine and epilepsy are two distinct neurological disorders that, at first glance, may appear unrelated. However, a growing body of research has unveiled comprehensive connections between these diseases. It may sound uncommon that migraine finds a place in the borderland of epilepsy, but this fact is substantiated by numerous pieces of evidence, including the fact that their distinction is often challenging [1]. Both migraine and epilepsy have diverse shared features, including a genetic component leading to a predisposed episodic pattern, resemblances regarding some pathophysiological mechanisms, as well as similarities in clinical manifestations and triggers [2–10]. Additionally, the array of antiepileptic drugs that are effective in both diseases, with some being used for migraine prophylaxis (e.g. Topiramate, valproic acid) represents another pillar supporting the existence of common pathogenetic mechanisms between migraine and epilepsy [2, 7, 11].

This review aims to delve into the existing knowledge regarding overlaps, similarities, and distinctions. It should serve as a resource for clinicians, as recognizing the commonalities and disparities in these disorders is crucial in providing accurate diagnoses and adequate treatment strategies. While other reviews have already been published on the connection between these diseases [2, 3, 12–21], this review focuses excessively on common and distinct clinical features throughout an attack or seizure, and on phenomena like peri-ictal headaches and clinical overlapping syndromes. Moreover, this review also considers the undoubtable interictal burden of individuals suffering from one or both diseases, providing insights into the challenges those affected face during and between attacks and shedding light on their cognitive and social abilities.

### Epidemiological aspects

Migraine occurs in approximately 1% of the population each year, with a 1-year prevalence rate of 11.7–13.2% [14]. The occurrence and distribution of epilepsy show global variations, but in Western countries, the annual new cases amount to approximately 0.05%. The prevalence varies from 0.4 to 1%, but the overall lifetime prevalence is considerably higher [14]. Although there is clear scientific evidence of an association between these two neurological conditions, the interpretation of epidemiological aspects can vary depending on factors encompassing study methodologies, diagnostic criteria, and patient characteristics including age [22, 23].

Recent epidemiological studies have indicated that individuals with epilepsy have a higher likelihood of suffering from headaches, especially migraines, compared to those without epilepsy. These headaches may be temporally unrelated to seizures, or they may occur before, during, or after an episode [1]. A significantly higher prevalence of headaches has emerged in female patients with epilepsy (63.0%) compared to males (33.3%) [24]. In children with epilepsy and migraine, ictal autonomic symptoms can be isolated, long-lasting, and may occur more frequently during seizures, while in adults, such manifestations (in both epilepsy and headache) are usually associated with other motor or sensory ictal signs and symptoms [22, 25]. Therefore, despite the limited number of studies [26–28], the situation takes markedly different forms in the paediatric population, as emphasized earlier, that is why ictal epileptic headache (IEH) is a phenomenon showing a higher prevalence in the pediatric epileptic population [10, 29, 30].

To further explore the higher prevalence of headaches in patients with epilepsy, Atalar et al. [31] recently performed a nationwide interview study including a total of 809 patients suffering from idiopathic or genetic epilepsy. In this cohort, 508 (62.8%) patients reported regular suffering from any type of headache while 444 (87.4%) of those stated that their headache had no time relationship with seizures. This leads to a prevalence of 54.88% of interictal headache in the study population (444/809). Moreover, the most common type of interictal headache was migraine (41.2%), followed by tension–type headache (13.7%). They further investigated the characteristics of headaches and epilepsy syndromes and identified three different clusters in their population. In Cluster 1, which was primarily associated with juvenile myoclonic epilepsy, mostly frequent and severe headaches indicative of a migraine-type primary headache were present. On the other hand, characterized by focal seizures and generalized tonic-clonic seizure epilepsy syndrome, Cluster 3 displayed shorter and nonthrobbing headaches, resembling tension-type headache. Moreover, there was an intermediary Cluster 2 which represented patients with generalized tonic–clonic seizure epilepsy syndrome and migrainous headache features, acting as a transition zone between Clusters 1 and 3. Another interview-based study by Osama A. et al. from 2022 compared two equal groups (a total of 62 subjects) of epileptic subjects with non-epileptic subjects regarding demographic data, clinical manifestations of epilepsy and headache as well as their temporal connection. They showed that primary headaches were more frequently seen in epileptic participants (61.3%) with migraine-type headaches being the second most encountered primary headache (12.9%) after tension-type headaches (45.2%).

The comorbid relationship between migraine and epilepsy might be explained by different theories: While the first theory states that migraine and epilepsy occur randomly in an individual, the observed prevalence of epilepsy in migraineurs and migraine in epileptics significantly exceeds expectations based on a simple random association [32]. In particular, the prevalence of epilepsy among those with migraine ranges from 1 to 17%, with an average of 5.9% [1], markedly higher than in the general population, where it ranges from 0.5 to 1% [22]. These data indicate a 52% increased prevalence of migraine among epileptic patients compared to non-epileptic individuals and a 79% increased prevalence of epilepsy among migraineurs compared to non-migraineurs [2].

Another theory suggests a unidirectional relationship, in which one condition predisposes the occurrence of the other. To illustrate it by way of example, migraine could lead to brain damage [33] which in return could make the brain susceptible to epilepsy. However, this hypothesis has not been widely confirmed, although there are controversial cases of “migralepsy,” in which migraine aura can trigger an epileptic seizure [32].

According to another theory, environmental factors, like head trauma, can increase the risk of both migraine and epilepsy [33]. Research has shown that people with epilepsy caused by head trauma have a higher risk of migraine. However, this explanation does not fully cover the increased risk of migraine in patients with idiopathic epilepsy, which is not linked to environmental causes [32].

The last theory is based on the possibility that genetic factors predispose a person to both conditions, as it seems that migraine is more common among relatives of epilepsy patients and vice versa. In some families with idiopathic temporal lobe epilepsy, there is a higher incidence of migraine. The most evident connection is observed between migraine with aura and epilepsy [33].

### Pathophysiological backgrounds - CSD and neurotransmitters

#### Cortical spreading depression

Cortical spreading depression (CSD) manifests as a neuronal and glial depolarization wave, which slowly spreads (3–5 mm/minute) over the grey matter and is accompanied by alterations in cerebral blood flow, ionic imbalance, and variations in the energy metabolism and neurotransmitter levels [34–36]. The possible activation of CSD due to subclinical epileptic discharge was first suggested by Parisi et al. [16]. CSD is hypothesized to be the fundamental mechanism behind migraine aura and multiple investigations have shown the interconnection of CSD and epileptiform ictal activities, which has provided evidence to suggest an epileptogenicity of CSD [37] that may explain the IEH concept. This thesis was further fueled by the introduction of the term “migraine aura triggered seizure” in the ICHD − 3 [38].

### Neurotransmitters

#### Glutamate

Glutamate, the main excitatory neurotransmitter in the human brain is believed to play a pivotal role in migraine pathogenesis. A meta-analysis examining the levels of migraine biomarkers in the cerebrospinal fluid and blood found elevated glutamate levels in the cerebrospinal fluid in episodic and chronic migraineurs [39]. Elevated levels of this neurotransmitter are believed to create an imbalance of excitatory and inhibitory activity in the brain which leads to a state of hyperexcitability [40]. This falls in line with the findings of a meta-analysis including studies using hydrogen magnetic resonance spectroscopy, as researchers found that multiple studies reported interictal abnormalities in excitatory and inhibitory neurotransmitters [40] A potential mechanism behind these abnormalities could be found in the ionotropic glutamate NMDA receptor (NMDAr). This receptor is hypothesized as a trigger to CSD by producing a self-sufficient current [41] as studies have shown that NMDAr inhibition can raise the threshold for CSD [42, 43]. Moreover, a recent study has revealed that NMDAr may primarily play a role in propagating CSD rather than initiating it [44]. Yet in another research, NMDAr has been considered essential for the initiation of CSD but exhibits a delayed response, which mirrors the timeframe of impaired glutamatergic clearance [45].

CSD is further suspected to not only be responsible for migraine aura but to be involved in the activation of trigeminal nociception and therefore triggering headache mechanisms. This thesis is supported by data demonstrating the ability of CSD to directly activate meningeal nociceptors [46]. However, the question if the activation of the trigeminovascular system by CSD is sufficient to trigger headache mechanisms remains a subject of differing opinions [47].

Aside from CSD, NMDAr also serves a pivotal role in initiating and maintaining peripheral and central sensitization which is the underlying pathological mechanism of allodynia. Animal models have shown that the local administration of glutamate can trigger this phenomenon, and the NMDAr blocker MK-801 or antagonists (±)-2-amino-7-phosphonoheptanoic acid effectively prevent the progression [48]. As a key excitatory neurotransmitter, the glutamatergic system participates not only in various stages of migraine pathogenesis but also in ictogenesis. NMDAr synthesis, location, function, and degradation are dynamic. Elevated glutamate levels and the occurrence of high-frequency oscillations have been noted preceding the initiation of an ictal event [49, 50]. Studies investigating electroencephalographic (EEG) recordings of ictal-like events have revealed preictal discharges are effectively inhibited by AMPA receptor (AMPAr) antagonists but not NMDAr antagonists [51]. These discoveries could enhance our understanding that AMPAr may have a more substantial role than NMDAr in terms of hyperexcitability during seizures.

#### GABA

Gamma-aminobutyric acid (GABA) is the main inhibitory signaling molecule that functions as a modulator of ionic channel current. Research indicated reduced occipital GABA levels detected by magnetic resonance spectroscopy in individuals experiencing severe and frequent migraine with aura episodes [52, 53]. However, more recent research conducted on individuals suffering from migraine with aura at a lower frequency has revealed no significant difference [54]. In another study investigating the relationship between the pathophysiology of migraine chronification and neurotransmitters, local GABA and glutamate/glutamine levels in the periaqueductal grey matter and dentate nucleus were examined [55]. Lower GABA levels in the dentate nucleus and higher glutamate/glutamine levels in periaqueductal grey matter in chronic migraine were found compared to the control group. These findings have been presented as probable markers for the chronification of migraine [55]. In contrast to the studies mentioned earlier, an alternate investigation utilizing a fully optimized sequence for GABA detection (in vivo H-magnetic resonance spectroscopy) confirmed elevated levels of GABA in migraineurs compared to healthy controls with no relationship between GABA levels and headache severity [56]. The Authors suggested that increased GABA concentration could be connected to the end of the previous migraine attack, before the current attack or the interictal phase. They have concluded that the condition might be due to vasodilation caused by GABA during CSD [57] or increased GABA as a result of neurogenic inflammation. Subsequently, another study using a similar methodology found a potential association between GABA metabolism and chronic pain [58]. The same authors have stated that this condition might not be a cause but an adaptive protective mechanism [59].

Seizures may also result from central nervous system overexcitation as a consequence of insufficiency of GABA. A magnetic resonance spectroscopy-based study indicated decreased levels of neuronal metabolites GABA in the thalamus, while there was an elevation in GABA and glutamine levels in the frontal lobe of juvenile myoclonic epilepsy patients compared to healthy controls [60]. Increased density of GABAergic neurons may be associated with an organizational defect of the cortex in juvenile myoclonic epilepsy [60]. In another study performed on patients with idiopathic generalized epilepsy, an increase in GABA and glutamine levels was observed, compared to healthy controls [61]. The diminished GABA receptor function could potentially be balanced by an elevation in GABA levels [62]. Elevated GABA levels might be due to compensatory mechanisms or the influence of medication [61]. Considering all this scientific data leads to the conclusion that there is a substantial interplay between glutamate and GABA regulation of both inhibition and excitation cycles in these two episodic paroxysmal disorders. Shared causative links will play a more prominent role in the future.

#### Genetics and channelopathies

Studies on migraine genetics have shown that migraine inheritance does not occur by the influence of a single gene but by the influence of many. The additional impact of environmental factors on the hereditary background leads to the definition of migraine as a complex genetic disease. There are well-known difficulties in identifying the genes that cause polygenic diseases, like migraine due to the low penetrance of the diseases, lack of detailed family histories, high phenotypic diversity, lack of disease-specific biomarkers, environmental factors with unclear effects and difficulties in modeling gene-gene relationships [63].

#### Genetics in migraine

The initial studies about genetics in migraine were conducted in the early 90’s [64, 65]. According to these studies, which mainly included twins and families, the relative risk for migraine of a first–degree relative of a proband with migraine without aura was up to threefold. These findings fuelled the belief of a strong hereditary background in migraine. Since then, years of research have brought further insights into the pathogenesis of this disorder. Due to great advances in technology, which enabled researchers to perform genome-wide association studies (GWAS), migraine is now believed to be a polygenic disease, in which the accumulation of many genetic variants, each having a small effect, leads to the occurrence [66].

GWAS have opened a new era in genetic research. These studies analyze several million SNPs (single nucleotide polymorphisms) covering the entire genome in a very large case and control cohorts enabling them can identify genes with small potency.

A recent meta-analysis, including 5 GWAS and 873.341 (102.084 cases and 771.257 controls) individuals of European ancestry identified 123 independent risk loci for migraine, 86 of which were previously unknown. The 123 risk loci were mapped to genes, and it was found that 80% of them contained at least one protein-coding gene within 20 kb. Moreover, the researchers found specific risk alleles for subtypes (migraine with and without aura) as well as evidence for the role of vascular and central nervous tissue types, proven by tissue enrichment analyses [67].

#### Genetics in epilepsy

Extensive gene sequencing studies have revealed the importance of gene mutations in the etiology of epilepsy. These data, combined with clinical genetic studies, confirm the notion that many forms of epilepsy are likely to have a genetic basis [68]. The results obtained with the mentioned genetic methods have moved scientists away from the idea of a single gene-single disease paradigm. Interestingly, both focal and generalized seizures can occur due to different expressions of the same genetic mutation. Moreover, there is clinical evidence for gene-environment interactions in epilepsy, however, many environmental factors have not been fully elucidated yet. There is substantial evidence supporting the role of gene-environment interactions in determining the likelihood of developing epilepsy following various brain injuries, such as traumatic brain injury and febrile convulsion. Today, epilepsies of unknown cause constitute a much smaller proportion, thanks to a better understanding of autoimmune epilepsies, neuroimaging, and the reclassification of many epilepsies previously thought to be idiopathic as having a genetic cause. A better understanding of the role of genetics in epilepsy has enabled us to witness the positive results of receiving a genetic diagnosis in terms of treatment selection and counseling [68, 69]. Moreover, it led to the detection of multiple genes that are highly associated with hemiplegic migraine but play a role in both disorders.

#### Hemiplegic migraine - the genetic bridge between certain forms of migraine and epilepsy

Hemiplegic migraine (HM) is defined as a subtype of migraine with completely reversible motor weakness, and additional visual, sensory, and/or speech symptoms [38]. Although motor symptoms usually last less than 72 h, they may persist for weeks in a minority of the patients. In familial hemiplegic migraine (FHM), at least one first or second-degree relative must experience hemiplegic migraine too. In sporadic hemiplegic migraine (SHM), although the patient himself meets the criteria for hemiplegic migraine, no first- or second-degree relative does [38].

New genetic studies and clinical data obtained from individuals suffering from FHM indicated the boundaries more clearly than in previous periods. Specific genetic subforms have been identified: In FHM type 1 (FHM1) mutations in the Calcium Voltage-Gated Channel Subunit Alpha1 A (*CACNA1A)* gene on chromosome 19 can be found. FHM type 2 (FHM2) disclosed mutations in the ATPase Na+/K + Transporting Subunit Alpha 2 *(ATP1A2)* on chromosome (1) Lastly in FHM type 3 (FHM3), there are mutations in the Sodium Voltage-Gated Channel Alpha Subunit 1 (*SCN1A)* gene on chromosome (2) The involvement of other loci that have not yet been identified may also be elucidated in future studies.

A recent review examined these genes involved in FHM and their association with the presence of epilepsy, together with clinical data [70]. This review included 28 families consisting of 195 individuals and evaluated mutations in *CACNA1A*, *ATP1A2*, *SCN1A*, and proline-rich transmembrane protein 2 (*PRRT2)* genes.

Of these 195 individuals, 78 suffered from epilepsy, 30 from focal and 30 from generalized epilepsy. When it comes to the underlying mutations in these individuals, *ATP1A2* mutation had the highest detection rate being present in 57.7%, followed by *PRRT2* (17.9%) *SCN1A* (16.7%), and *CACNA1A* (7.7%).

In the abovementioned study, researchers also found that the underlying mutations play an important role in whether patients suffer from FHM, epilepsy, or both. They examined the underlying mutation of all study participants and what diseases they suffered from. What they found was that five of the eight persons having a mutation in the *CACNA1A* gene suffered from epilepsy and FHM together, whereas one patient suffered from epilepsy only. The largest group, the one of those carrying *ATP1A2* gene mutations, consisted of 132 individuals from 15 families. Of these 132 persons, 33 had epilepsy and FHM together, while 12 had epilepsy alone.

There were 33 individuals included in the *SCN1A* gene group, 10 of which had epilepsy and HM together, whereas 3 only suffered from epilepsy.

Of the 22 patients with mutations in the *PRRT2* gene, nine patients had epilepsy and HM together, and five patients had only epilepsy. Ten patients of this latter group had self-limiting familial infantile epilepsy.

It is important to note that in all subgroups, epilepsy appeared before the occurrence of HM. When the EEG data of a total of 39 patients were evaluated, 51.2% had epileptic activity and 30.8% had slow wave activity (usually focal, rarely generalized); On the other hand, 18% had normal EEG findings. Epileptic activity was mostly localized in the temporal region, followed by the occipital, frontal, and parietal regions [70]. .

### Genetic mutations linking migraine and epilepsy

#### CACNA1A

*CACNA1A* on chromosome 19p13 was identified through positional cloning and mutation analysis of candidate genes in the pedigrees of multiple FHM families [71]. The *CACNA1A* gene encodes the α1 subunit of neuronal voltage-gated Cav2.1 (P/Q type) channels, which are predominantly localized to the presynaptic terminals of brain and cerebellar neurons and play an important role in the control of neurotransmitter release [72].

More than 25 pathogenic variants in *CACNA1A* with autosomal dominant inheritance have been reported for FHM1. They often have gain-of-function effects, causing increased calcium (Ca 2+) influx, which in turn leads to increased glutamatergic neurotransmission and neuronal hyperexcitability [73].

It is well known that *CACNA1A* mutations can also cause two other neurological disorders: episodic ataxia type 2 and spinocerebellar ataxia type 6. Episodic ataxia type 2 is characterized by attacks of paroxysmal ataxia, vertigo, and nausea, while spinocerebellar ataxia type 6 is characterized by adult-onset, slowly progressive cerebellar ataxia, dysarthria, and nystagmus [74, 75]. On the contrary, data regarding epilepsy is poor and the precise prevalence of epilepsy in individuals with *CACNA1A* mutation is unknown. However, a recently performed multicentric study found that individuals with *CACNA1A-associated* epilepsy mostly suffer from permanent cerebellar dysfunction and early moderate to severe global development delay and intellectual deficiency [76].

#### ATP1A2

The *ATP1A2* gene, located on chromosome 1q23.2, was identified as the second major FHM gene. The *ATP1A2* gene encodes the α2 isoform of the catalytic subunit of the Na+/K+-ATPase ion transport pump, which is responsible for the regulation of electrochemical gradients across cell membranes of the central nervous system, cardiac, skeletal, and smooth muscle tissues [77].

The Na+/K+-ATPase ion transport pump is expressed in astrocytes at synapses in the central nervous system, which functions in the clearance of extracellular potassium (K+) and production of the sodium (Na+) gradient used in the reuptake of glutamate. *ATP1A2* mutations contribute to FHM pathophysiology by increasing the propensity for CSD due to this mechanism. Thus *ATP1A2* mutations affect glutamatergic neurotransmission, causing the defective regulation of the balance of excitation and inhibition in the brain as seen in migraine [78, 79].

Individuals with *ATP1A2* mutations have a wide clinical spectrum, which includes alternating hemiplegia of childhood, epilepsy, mental retardation, behavioral problems, learning disability, ataxia, tremor, nystagmus, neuromuscular periodic paralysis disorders, recurrent coma, and fever.

More than 80 variants have been associated with FHM2, and approximately 25 of these variants have been diagnosed in sporadic cases. This high rate indicates that de novo mutations are common in the *ATP1A2* locus [77].

*ATP1A2* mutations have also been shown to be effective through the mechanisms of changing the K + sensitivity of the pump, reducing the Na+/K + turnover rate, and generating non-functional proteins [63].

#### SCN1A

*SCN1A* gene on chromosome 2q24.3 was identified as causative for FHM3, which is rarer than FHM1 and FHM2. *SCN1A* mediates the voltage-dependent sodium ion permeability of excitable membranes (primarily the inhibitory GABA-ergic interneurons) of the central nervous system via the α1 subunit of the neuronal voltage-gated sodium channel Nav1.1 [72].

A GWAS including 1018 individuals with mesial temporal lobe epilepsy and hippocampal sclerosis and 7552 control subjects revealed a significant association for mesial temporal lobe epilepsy and hippocampal sclerosis with febrile seizures at the Na + channel gene cluster on chromosome 2q24.3. These findings suggest that *SCN1A* might be involved in this syndrome, providing a new aspect of mesial temporal lobe epilepsy and opening horizons for the investigation of prognostic factors and prevention of epilepsy in a certain part of children with febrile seizures [80].

#### PRRT2

As another important gene, variations in the *PRRT2* gene have been shown to cause a variety of diseases, including benign familial infantile epilepsy and paroxysmal kinesigenic dyskinesia. Next-generation sequencing techniques have allowed the broadening of this disease spectrum. *PRRT2* gene variants may also cause FHM in some patients, suggesting that it may be a fourth gene underlying FHM [81].

*PRRT2* interacts with the synaptic target Soluble N-ethylmaleimide-sensitive-factor attachment protein receptor, probably playing a role in synaptic vesicle machinery and neurotransmitter release. The affected protein is predominantly expressed in neurons and neuroendocrine cells and is concentrated in presynaptic terminals, regulating Ca2+-triggered exocytosis in synaptic vesicles [82]. Recent studies have shown that *PRRT2* causes negative modulation of voltage-gated Nav1.2 and Nav1.6 channels [83].

Enhanced neurotransmitter release from excitatory synapses or decreased release of inhibitory neurotransmitters is thought to lead to a state of hyperexcitability. This situation may predispose to basal ganglia dysfunction and deterioration in cortical circuits, resulting in *PRRT2*-related paroxysmal disorders. These dynamics, which depend on excitatory and inhibitory synapses rather than basal synaptic transmission properties, may explain the paroxysmal nature of dyskinesia elicited by a kinesigenic trigger alone.

Although *PRRT2*-associated diseases are not considered “channelopathies” in the classical sense, but rather synaptopathies, their similarities with episodic diseases associated with mutations in ion channels are evident.

Interestingly, among *PRRT2* mutation carriers, out of 1444 patients, 32 patients had migraine with aura, 36 patients had migraine without aura, and 34 patients had HM [82] (Table 1).

**Table 1:** Genes involved in migraine and epilepsy genesis (The table should be placed at the end of the chapter “Genetic mutations linking migraine and epilepsy”)

Gene	Function	Gene locus	Associated Conditions
CACNA1A	Regulation of Ca 2+ - channels	19p13	FHM 1, episodic ataxia type 2, spinocerebellar ataxia type 2, epilepsy
ATP1A2	Regulation of Na+/K+-ATPase	1q21-q23	Wide spectrum: i.a. FHM 2, epilepsy, mental retardation,
SCNA1	Regulation of Na+ - channels	2q24	FHM 3, mesial temporal lobe epilepsy, and hippocampal sclerosis
PRRT2	Regulation of Ca2+ - mediated neurotransmitter release and voltage-gated ion channels	16p11.2	Wide spectrum: i.a. FHM 4, benign familial infantile epilepsy, and paroxysmal kinesigenic dyskinesia

#### Common and distinct clinical features

Migraine and epilepsy share several clinical features, making distinguishment for clinicians difficult in some cases. These similarities and shared features are not only found in the different phases of a migraine attack and an epileptic seizure as peri-ictal headache are seen frequently. This chapter aims to shed light on the similarities and differences that occur during the ictal and peri-ictal phases. Moreover, it will describe the different types of seizure-related headaches and put some focus on clinically overlapping syndromes and disorders that can mimic migraine and epilepsy.

#### Trigger factors

Trigger factors are, regardless of the disease, defined as endogenous (f.e. menstruation) or exogenous (f.e. weather) agents reducing the threshold of an attack [84]. Many individuals suffering from migraine or epilepsy report a variety of trigger factors. Especially in migraine, these factors are not generalizable and show high diversity between individuals. A study [85] including 1207 individuals with migraine, of which 85% were female found that about 75% of migraineurs reported to have trigger factors, with a mean number of 6.7 trigger factors per patient. The most common trigger factors in migraine were stress (79.7%), hormones (65.1%), missing a meal (57.3%), weather (53.2%), and sleep disturbances (49.8%). Other factors reported encompassed smelling perfume or odor (43.7%), neck pain (38.4%), lights (38.1%), alcohol consumption (37.8%), heat (30.3%), certain types of food (26.9%), exercising (22.1%) and sexual activity (5.2%). However, some of the most common trigger factors in migraine like bright light or sleep deprivation are contentious as they could depict premonitory symptoms of an already ongoing attack and are mistaken for triggers by the affected individuals [86]. When it comes to trigger factors in epilepsy, a similar pattern can be observed. A prospective study performed in 2013 which included 104 patients found that 97% of study participants reported the presence of at least one trigger factor for an epileptic seizure. Other sources reported the prevalence to range from 50 to 90% [87–89]. The most common factors in this study were stress (82%), sleep deprivation (71%), fatigue (68%), and poor compliance with antiseizure – medication (54%). Furthermore, menstrual cycle, emotions, alcohol, fever, and others were reported by the participants. Interestingly, a high number of similarities can be detected when comparing the triggering factors of migraine and epilepsy. In both diseases, stress is the number one triggering factor, followed by sleep disturbances, hormonal factors, alcohol, illness, and other stressors.

What most of these factors have in common is that they can be seen as alterations in daily activity or environment. Moreover, it is assumed that, especially in migraine, the combination of multiple trigger factors is more potent and might be necessary in some individuals to trigger an attack [90]. However, the role of trigger factors in the genesis of a migraine attack or an epileptic seizure is controversial. It is a difficult challenge to genuinely identify attack triggers, as this is mostly done in retrospective studies which are limited by recall bias and false attribution [91]. Moreover, some of the most common trigger factors in migraine like bright light or sleep deprivation could depict premonitory symptoms of an already ongoing attack which are mistaken for triggers by the affected individuals [86].

It is undisputed, that migraine and epilepsy have a multifactorial nature of cause. Therefore, it seems plausible that the brains of those affected are susceptible to shared influences. Factors like stress or illness depict a disruption of the balance in humans and could therefore contribute to the activation of pathways leading to these neurological events. However, their role is highly speculative, and further research is warranted.

**Table 2:** The most common trigger factors in migraine and epilepsy (The table should be placed at the end of the subchapter “Trigger factors”)

Migraine attack triggers	Seizure triggers
Stress	Stress
Sleep deprivation	Sleep deprivation
Missing a meal	Fatigue
Changes in weather	Poor compliance with Antiseizure medication
Hormones	Hormones

#### Auras

Aura is a phenomenon that occurs in both disorders and is characterized by reversible neurological symptoms. Around 20% of people with migraine experience auras [92]. Migraine aura can last from 5 to 60 min including mostly visual phenomena, paraesthesias or speech difficulties, and in rare cases brainstem disturbances [52]. Epileptic auras are very diverse, showing a wide range of symptoms that can manifest in every major sense. They are usually short-lasting, from a few seconds to a few minutes, however, there are also cases of “aura continua” described. Those auras pertaining to the primary senses can be divided into olfactory, gustatory, visual, somatosensory, and auditory. This subdivision becomes more complex when it comes to the auras concerning higher-order processes, emotions, and autonomic alterations, which lead to a greater number of variations. Moreover, the symptoms during aura depend on the type of epilepsy. While individuals suffering from absence epilepsy often experience dizziness, loss of time, and spaciness feelings, those who suffer from myoclonic epilepsy report symptoms like tingling, electricity, and the feeling of shocks. When it comes to focal epilepsy, frequently reported auras encompass epigastric sensation, visual hallucination, and other sensory perceptions [93].

For clinicians, it has been difficult ever since to distinguish between visual aura in migraine and focal epileptic seizures followed by post-ictal headache (PIH). Nevertheless, there are some clinical differences (Table 2). While visual aura in migraine can last from five minutes to one hour, the typical duration of epileptic aura ranges from a few seconds to a few minutes. However, there are cases in which long-lasting visual aura was described and diagnosed using EEG [94]. Other factors facilitating distinguishment are the onset of symptoms, the visual field, and the presence of accompanying symptoms. The onset of symptoms is usually very sudden in epilepsy while it is slowly progressing in migraine. Moreover, the visual field shows interesting differences: Visual aura in epilepsy is characterized by restriction to one hemifield with stereotypic affection to this location presenting round or point-like phenomena. On the contrary, the centrifugal or centripetal spread can be only observed in migraine, often looking like a zigzag pattern. Clinicians should also search for the presence of other symptoms which are migraine or epilepsy–like (like headaches or seizures) and can help distinguish the two auras [95, 96] (Table 3).

**Table 3:** Clinical characteristics of visual aura in migraine and epilepsy (The table should be placed at the end of the subchapter “Auras”)

	Migraine	Epilepsy
**Duration**	> 5 min	< 5 min
**Onset of symptoms**	Slowly progressing	Sudden
**Accompanying symptoms**	Headache, Nausea and Vomiting, Photo- and Phonophobia	Seizures, other auras
**Visual field**	Showing centrifugal or centripetal spread	Restricted to one hemifield

#### Attacks and seizures

Both diseases exhibit attacks that can be divided into 4 parts: the prodromal phase, the aura phase, the pain or seizure phase, and the postdrome or postictal phase.

During the prodromal phase of migraine, the person affected can have a feeling of neck pain, tiredness and yawning, dizziness, and experience concentration difficulties [14]. On the other hand, prodromes in epilepsy, including focal and generalized seizures, can last for hours and days and occur as a change in mood, behavior, and demeanor or more discrete symptoms like headache, dizziness, restlessness, or tiredness [93].

The headache or pain phase in migraine is characterized by headache with moderate to severe intensity, which is unilaterally located and has a pulsatile character. During this period, nausea, vomiting, and/or photo- and phonophobia are often experienced. In the great majority of cases, migraine attacks last between 4 and 72 h, however, in rare occasions the 72-hour limit is exceeded, which is defined as “status migrainosus” [38]. In this case, an EEG should be performed to rule out epileptic headache and ensure the diagnosis is accurate [25] (Table 4).

**Table 4:** Headaches and their characteristics in cranial and vascular disorders. (The table belongs to the chapter “Migraine and epilepsy mimics in neurovascular disorders”)

Vascular disorder	Headache characteristics	Seizures
Ischaemic stroke	Moderate, diffuse, unspecific, posterior circulation	6–8% Possible
TIA	Neurological deficits can mimic migraine aura, headache simultaneous to deficit	Limb-shaking (non-epileptic)
Non-traumatic intracerebral haemorrhage/SAH/ASDH	Sudden onset/thunderclap headache	Possible
Unruptured aneurysm	Can mimic migraine and other primary headaches, worsening while growing	Possible
AVM	Uncommon, it can present as migraine with aura	Usual
DAVF	Diffuse headache, worse in the morning, coughing and bending over, associated with pulsatile tinnitus +/- ophthalmoplegia	Possible
Cavernous angioma	Usually infrequent, unspecific, and secondary to cerebral hemorrhage, and/or seizures	Usual
Sturge-Webber syndrome	Migraine-like headache	Usual
GCA	New persistent headache > 60 years old, Amaurosis fugax, jaw claudication	Unlikely
PACNS or SACNS	Unspecified headache	Usual
Cervical, vertebral or intracranial dissection	Unilateral pain, without typical migraine features	Unlikely
Post-endarterectomy, angioplasty, or stenting	Mostly diffuse and mild to moderate, can be unilateral without typical migraine features	Possible
CVT	Intracranial hypertension headache	Possible
Cranial venous stenting	Ipsilateral to stenting	Unlikely
Angiography/endarterial procedure	Can cause triggered migraine	Unlikely
RCVS	Sudden/thunderclap headache	Yes
CADASIL	Migraine with aura	Possible
MELAS	Migraine-like headache	Usual
MMA	Migraine-like headache	Possible
CAA	Migraine-like, aura	Possible
RVCLSM	Migraine-like headache	Yes
Pituitary apoplexy	Sudden headache, visual loss	Unusual

Opposed to that, there is a great number of variations in the characteristics of epileptic seizures. A seizure is defined as a “transient occurrence of signs or symptoms due to abnormal excessive or synchronous neuronal activity in the brain that usually lasts less than 2 minutes”. The International League against epilepsy (ILAE) classifies seizures based on onset, awareness, and the presence of a motor component. The onset can be further divided into focal, generalized, unknown, or unclassifiable onset and the second factor, awareness during the seizure, can be divided into impaired or not impaired [97]. Status epilepticus is a condition resulting either from the failure of the mechanisms responsible for seizure termination or from the initiation of mechanisms, which leads to abnormally, prolonged seizures [98]. Based on this classification, the significant variability in the clinical presentation of epileptic seizures becomes apparent. The postdromal or postictal phase in both diseases is the least studied and understood phase. Although being mostly characterized by non-headache/non-epileptic symptoms, they are often very disabling for the affected individuals, leading to a prolongation of symptoms experienced due to a migraine attack or epileptic seizure [99].

The symptoms experienced by migraineurs during the postdrome phase can be divided into neuropsychiatric, sensory, gastrointestinal, and general symptoms. The most common of those are tiredness, concentration difficulties, and neck stiffness, but also light sensitivity, thirst, and constipation have been reported. The average duration of the postdromal symptoms in migraine lies between 18 and 25 h [99].

The duration and clinical features of the postictal phase in epilepsy show, similar to the seizure itself, a wide variety. The duration of postictal deficits depends on age, type of seizure, and underlying brain disease, leading to a range from a few minutes in focal epilepsies to one or two days, as seen in Todd’s paresis. The symptoms can be divided into subgroups, encompassing altered consciousness, cognitive dysfunction, autonomic dysregulation, headache, changes in mood and affect, postictal paresis, psychiatric symptoms, language dysfunction, and others, with the most common symptom being unresponsiveness [100].

#### Migrainous features in seizure-related headache

The co-occurrence of headaches and epileptic seizures has been addressed more frequently from a temporal perspective, thus, categorizing the headaches as manifesting before, during, after, or completely unrelated to epileptic seizures. Interictal headaches are defined temporally as occurring more than 24 h before or 72 h after the epileptic event, while peri-ictal headaches occur a short time before, throughout, or shortly after the seizure. Taking into consideration the possibility of the overlap between the above-classified headaches and epilepsy (seizure manifestations), attentiveness to the clinical manifestations and their temporal relation is mandatory for assuring suitable diagnosis and treatment. Noteworthy, the ILAE classification does not address headaches in relation to epileptic seizures, in contrast to the International Classification of Headache Disorders 3rd edition (ICHD-3) [3, 4].

 According to the ICHD-3, headaches with a temporal connection to seizures (seizure-related headaches) are classified in two separate sections as follows: IEH (Sect. 7.6.1) and PIH (Sect. 7.6.2) are attributed to epileptic seizures, while migraine aura-​triggered seizure is considered to be part of migraine complications (Sect. 1.4.4) [3]. A 2023 study by Schiller et al. reported seizure-related headaches in around 40% of epileptic patients suffering from headaches, with the majority suffering from post-ictal headaches [101]. Another recently published paper by Ekizoglu et al. [102] which collected data from a large cross–sectional study further investigated the clinical associations between peri-ictal headache and epilepsy. They found that peri-ictal headache was present in 13% of patients, showing a higher prevalence in females and those reporting a family history of epilepsy or migraine. Moreover, a highly interesting finding was that the presence of peri-ictal headache is associated with a poorer outcome in epileptic treatment showing lower rates of seizure freedom over five years and higher rates of drug resistance and use of polytherapy.

Another noteworthy aspect to be considered when addressing the migrainous features of seizure-related headaches was shown in one study by Scutelnic A et al., namely that some of the typical manifestations of migraine aura and migraine headache diminish with increasing patient age, thus influencing the diagnosis of migraine aura in senior patients [103]. Additionally, considering, on one hand, the subjectivity of headache description by the patient and on the other hand the relation of epileptic seizures with impaired memory, a clear limitation on proper reporting of headaches is present [3].

#### Pre-ictal headaches

Considered controversial by the ICHD-3, pre-ictal headaches are defined as occurring under 24 h and until the onset of the seizure in the absence of epileptic activity on EEG recordings. The controversy around their existence stems from studies where the definition of pre-ictal headaches was not accompanied by a report of EEG recordings, thus not being able to be differentiated from ictal headaches [3]. Nevertheless, although not categorized, further studies are recommended in the ICHD-3 to establish the existence, characterization, and prevalence of this type of headache [38]. Existing studies on pre-ictal headaches diagnosed clinically place their incidence in epileptic patients between 1 and 10%, from which up to more than half are characterized as migraine-like. A study using video EEG showed that 3% of the epileptic study population reported headaches without EEG activity before the seizure [2–4].

#### Ictal epileptic headaches - IEH

The concept of IEH was first coined by Parisi P in 2012 [25] and finally incorporated in the appendix section of the ICHD III classification. IEH are defined as headaches occurring at the same time as the onset of a focal seizure, while either improving or remitting after seizure termination and/or being ipsilateral to the epileptic focus [38]. The ILAE classifies IEH as a “focal sensory seizure with cephalic sensation”, meaning a sensation in the head such as light-headedness or headache where “cephalic aura” covers a large variety of signs and symptoms related to the head. Some of these are light-headedness, dizziness, numbness, pain, pressure, electrical shock-like sensations, etc. [2]. . Furthermore, cephalic aura can be seen mostly in frontal lobe epilepsy and more rarely in temporal and parieto-occipital epilepsy [2, 6–8, 11, 104]. Headache as the sole ictal epileptic event without any other accompanying symptoms or signs was first documented and published in 2007 [105]. This ictal event was recorded with an ictal EEG, and the remission of the headache and the EEG abnormalities by i.v. admission of diazepam was also demonstrated.

Accordingly, to the numerous and different types of headache, it is very likely that the cortical projections of headache pain are widespread projections, thus implying areas that are also involved in the autonomic network system (insula, cingulate cortex, prefrontal cortex, amygdala, and other parts of the limbic system) and not just the primary sensory-sensitive areas. So we may consider at least some cases of IEH as “autonomic seizures” [9, 10, 30].

In particular, Parisi P et al., suggested the possibility of considering IEH as an autonomic seizure, because of the behavioral similarity showing and comparing IEH with the autonomic seizures in Panayiotopoulos Syndrome (a self-limiting focal epilepsy of childhood characterized by rare but prolonged focal autonomic seizures) with consequent long-lasting duration, without involvement or diffusion to other cortical areas (as often reported in these patients, in pediatric age), In this regards, Parisi P et al. first suggested the possible activation of CSD due to “subclinical” epileptic discharge to explain the IEH concept - headache as the sole ictal epileptic manifestation [9, 10, 16, 17].

IEH has various clinical presentations with patients describing migraine-like features followed by tension-type or atypical features. Regarding localization, the headache can be diffuse or lateralized [2, 25]. The headache features are variable and do not always relate to lesion localization or epileptic focus on EEG [104]. In other words, there is a lack of a clear repetitive EEG headache-associated pattern. The ictal EEG recording in most IEH patients does not yield a particular EEG pattern, nor specific cortical correlations (e.g. focal frontal, parietal, temporal, occipital, and primary or secondary generalized), as it has also been reported for autonomic manifestations in children affected by Panayiotopoulos syndrome, showing both ictal autonomic manifestations and ictal epileptic headache.

Although usually lasting seconds to minutes, the duration of IEH can vary and go up to hours in the case of nonconvulsive status epilepticus [2]. Nevertheless, a short duration of the headache episode can be suggestive of ictal headache with a study reporting IEH in patients with a duration of < 35 s. Migraine-like features include photo- and phonophobia, vomiting, nausea as well as irritability, pallor, agitation, or speech difficulties [8, 11, 104].

All in all, IEH are heterogeneous in their clinical presentation, EEG patterns, specific cortical localizing relationships as well as duration [8].

#### Post-ictal headaches

Among the peri-ictal headaches, post-ictal headaches (PIHs), caused by epileptic seizures, are considered to have the highest prevalence, thus occurring in a significant proportion of epileptic patients. The timing of the headache is characterized by an onset of < 3 h after seizure cessation and remission < 72 h after the end of the seizure [2, 3, 5, 38].

Studies have shown that about 50% of individuals with PIHs experience migraine-like headaches, with a meta-analysis reporting a third of epileptic individuals experiencing PIHs and 16% post-ictal migraine [2, 3, 5]. Additionally, studies have shown that occipital epilepsy had a higher incidence of PIH compared to temporal and frontal epileptic focus, the same applies to convulsive versus non-convulsive seizures. Post-epileptic headaches occur mostly after bilateral tonic-clonic seizures [11].

Moreover, PIHs with migrainous features were reported in several studies with up to 48% of adult patients reporting them [6].

An important aspect of PIHs is represented by the reduced attention received, with both neurologists and patients focusing mainly on the seizures and leaving out the headache treatment with analgesics, leading to low rates for the use of analgesic medication. In many cases, the encountered migraine-like features might not respond to simple analgesics like paracetamol or non-steroidal anti-inflammatory drugs, requiring more specific treatments like triptans [2, 3, 104].

#### A critical view on the entity “migralepsy”

Migralepsy, a compound word of the terms “migraine” and “epilepsy”, was first described by Lennox and Lennox in 1960 [106]. Not to be mistaken with ictal epileptic headache, which refers to headache being the main symptom of a focal epileptic seizure, migralepsy describes the unlikely event of migraine aura being followed by an epileptic seizure within one hour [2]. However, this entity is highly controversial due to several reasons.

Migralepsy was listed in the ICHD – 3, under the term “migraine – aura triggered seizure” [38]. According to this classification, the diagnosis can be made when an epileptic seizure, which fulfills the criteria for at least one type of epileptic attack, occurs during or within one hour after a migraine with aura attack [38].

The decision to list migralepsy in the IHS classification was, however, subject to different opinions [107] The main criticism was that the definition of this condition would be inadequate or too narrow, which could promote misdiagnosis [108, 109].

The risk of misdiagnosis was also shown by Hartl et al. [95] when they examined the differences between the characteristics of visual aura in migraine and epilepsy. They found characteristics typical for either migraine or epilepsy, concerning duration, lateralization, visual sensations, presence of headache, and accompanying symptoms. Nevertheless, they stated that the differentiation can indeed be very difficult and suggested to include further features in the classification.

The likelihood of misdiagnosis was also confirmed by Sances et al. [110], when they performed a literature search finding 50 cases reported as migralepsy. These cases were reviewed systematically, concerning diagnostic criteria, symptoms, EEG findings, and uncertainty of described information. They found that only 2 cases supported the criteria of migralepsy, while 19 were classified as uncertain because of the limited information available and 14 were highly likely to be occipital lobe seizures.

Supported by these facts, some authors gained attention by claiming to detach migralepsy from the International Classification of Headache Disorders (ICHD) [110], while others stated that, in their opinion, the “migralepsy concept” wouldn’t exist at all and that most migralepsy cases would be epileptic events with ictal headache or visual aura being the only clinical features [107, 111].

These arguments are fuelled by the fact that the pathophysiology behind migralepsy is still unclear. However, there are a few hypotheses about possible mechanisms. One very common hypothesis states that the hyperexcitability of the cortex found in migraineurs and making the cortex susceptible to CSD, the basis of migraine aura, could be a trigger for epileptic seizures in people suffering from epilepsy [112, 113].

As this thesis could explain the pathophysiology of migralepsy, it seems questionable why it doesn’t affect a higher proportion of people. Following the numbers of the Global Burden of Disease study [114], at least 1–2 in 1000 people may suffer from both, migraine and epilepsy and therefore should also be susceptible to migraleptic attacks. However, confirmed cases are very rare.

To find out more about this entity and whether it deserves to be listed in the ICHD–3, further research is warranted. Verroti et al. claimed to have all subjects undergoing an ictal EEG who display features of both conditions, migraine, and epilepsy, to find out the underlying mechanisms of these episodes and enable a reliable diagnosis. This would also contribute to solving the question of whether migralepsy is truly what it appears to be [107].

#### Clinically overlapping syndromes

Besides the peri-ictal occurrence of headache and migraine in epilepsy, other syndromes are strongly associated with migraine and epilepsy. While familial hemiplegic migraine was already discussed in the genetics chapter, childhood epilepsy syndromes, and mitochondrial disorders also may present in an individual with seizures and migraine attacks.

#### Childhood epilepsy syndromes

Childhood epilepsy syndromes mainly affect children between 2 and 12 years of age, although some syndromes have a larger variability regarding the age of onset [115]. The most popular childhood epilepsy syndromes are Gastaut syndrome, self-limited Epilepsy with Centro-Temporal Spikes (SeLECTS), and panayiotopoulos syndrome.

Childhood epilepsy syndromes may often exhibit common features reminiscent of migraine [116, 117] leading to challenges in differentiating the two distinct paroxysmal conditions [116, 118, 119]. Nevertheless, the patient’s history, clinical manifestations, and EEG changes aid in their distinction. Both migraine and childhood epilepsy syndromes have a favorable prognosis, but the treatment goals differ considering the mostly remissive course of childhood epilepsies [115, 116, 118] in contrast to migraine where complete absolution is not expected.

Migraine and childhood epilepsy both have a high prevalence in the paediatric population [118] with a retrospective study by Toldo et al. reporting on the existence of a connection between the two disorders. According to this study, the presence of migraine is associated with a higher risk of epilepsy and vice versa [28]. Other studies further emphasized the relation between epilepsy in migraine, one revealing a higher risk for children suffering from migraine with aura developing unprovoked seizures in contrast to those without aura [117] and others showing that a quarter of the epileptic children were also migrainous [120–122].

Furthermore, similar to adults, next to peri-ictal headaches, post-ictal headaches are the most frequently encountered with some of them having migraine-like features [116–118]. In childhood epilepsy syndromes, postictal headache is seen in up to 40% of children with self-limited focal epilepsies. The risk of migraine is also seen in relatives of children affected by benign rolandic epilepsy [118].

A wide array of conditions acts as predisposing factors for both childhood epilepsy and migraine, from traumatic brain injury, brain tumors, infections, and vascular disorders of the central nervous system to genetic and metabolic diseases such as Sturge-Weber syndrome, tuberous sclerosis complex, alternating hemiplegia, etc. [118]. .

Self-limited epilepsy syndromes have several common characteristics: their occurrence is age-related, in children without any remarkable history of disease or pathological changes in cognition and neurological examination. Each syndrome is characterized by typical semiology for the seizures as well as typical EEG features, frequently identified during sleep, while maintaining a normal EEG background. Additionally, their cause remains unknown, despite an observed higher incidence of positive family antecedents of epilepsy and genetic predisposition for the EEG modifications. Moreover, all of these epilepsies have a good response to medication.

#### Mitochondrial encephalomyopathy, lactic acidosis, and stroke-like episodes - the MELAS Syndrome

MELAS is a rare mitochondrial disorder characterized by a wide array of clinical features, due to the multi-organ involvement [123]. The cardinal feature of the disease is stroke-like episodes associated with encephalomyopathy, lactic acidosis, seizures, and a wide array of other clinical signs and symptoms [120, 123, 124]. The seizures and stroke-like episodes observed in MELAS often bear a resemblance to migraine with aura. They frequently encompass visual disturbances, hemianopsia, cortical blindness, as well as other signs and symptoms of central nervous system implication (hemiparesis, deafness, ataxia, exercise intolerance, muscle weakness, etc.). Headaches occur in most affected patients, resembling migraines due to increased severity, pulsatile character, and association with vomiting. These migraine-like headaches occur recurrently and are a typical characteristic of MELAS that can trigger stroke-like episodes [123] further complicating the differentiation between the two disorders [120]. Frequently the peak of severity for these headaches occurs during stroke-like episodes. Nevertheless, MELAS is diagnosed using genetic testing, namely by identifying the specific genetic mitochondrial deoxyribonucleic acid mutation, namely the m.3243 A > G in the MT-TL1 gene as well as the cardinal elements of the disease. The treatment for both disorders is different, with migraines being treated with lifestyle changes and medications, while MELAS requires multidisciplinary, symptomatic, and supportive treatment [120, 123, 124].

#### Migraine and epilepsy mimics in neurovascular disorders

Various cerebrovascular diseases may present with migraine-like features and lead to seizures either at the beginning or throughout the disease.

### Disturbed cerebral blood flow – hypoperfusion

#### Ischaemic stroke

Ischaemic stroke may present with headache in up to 30% of cases, more commonly in posterior cerebral circulation [125].

During an ischaemic stroke, oligohaemia may lead to cell hypoxia and hypoglycemia, and consequently excitotoxicity (sudden release of neurotransmitters due to brain cell death). Those disturbances can produce an irritative effect to adjacent cells which can lead to the presentation of seizures in the acute phase of the stroke. Post-stroke seizures occur in 6–8% of ischaemic strokes. A recent cohort multicentral study observed that a higher risk of seizures was found in larger strokes and strokes located in the Posterior Cerebral Artery, and reperfusion treatment was not associated with acute symptomatic seizures [126].

#### Genetic and acquired angiopathies

In addition to acute cell damage due to ischaemic stroke, genetic and acquired angiopathies can also mimic migraine episodes and epilepsy.

In genetic vascular conditions such as MELAS and Cerebral Autosomal Dominant Arteriopathy with Subcortical Infarcts and Leukoencephalopathy (CADASIL), chronic small vessel hypoperfusion is seen [123, 127]. In MELAS, mitochondrial dysfunction can lead to angiopathy and nitric oxide (NO) deficiency, therefore ischaemic insults are seen in the brain tissue [123] and present typically with migraine-like headaches and seizures [128]. CADASIL is a genetic vasculopathy caused by the mutation of the NOTCH3 gene that leads to NOTCH3 protein accumulation in the blood vessel wall, hence the thickening of the blood vessel wall and therefore chronic small vessel hypoperfusion [127].

Encephalotrigeminal or leptomeningeal angiomatosis (Sturge-Weber Syndrome) can present as migraines-like attacks with prolonged aura, seizures, cognitive impairment, and cerebral atrophy. Neurologic symptoms appear due to chronic hypoperfusion and hypometabolism, probably due to the heightened constrictor tone of cortical vessels [129]. This condition may trigger seizures, brain atrophy, and in some cases migraine-like auras, while headaches are more often seen as post-seizure headaches [38].

Retinal vasculopathy with cerebral leukoencephalopathy and systemic manifestations (RVCLSM) is a systemic blood vessel disease caused by mutations in TREX1, leading to chronic ischemia and as a consequence neurological deficits, cognitive impairment, psychiatric disturbances, seizures, migraine-like headaches and systemic manifestations [38].

Hypoperfusion is presumably also one of the causes of headaches and seizures if brain damage coexists in cerebral amyloid angiopathy [130], Moya-Moya angiopathy [131], giant cell arteritis [132], and angiitis of the central nervous system (PACNS/SACNS) [133] due to narrowing of brain arteries.

#### Disturbed cerebral blood flow – hyperperfusion

Reperfusion techniques in a chronically ischaemic brain can result in cerebral hyperperfusion syndrome, a life-threatening condition that may begin as severe migraine-like headache which may progress to seizures and coma. The presence of vasodilators in response to chronic hypoperfusion can increase cerebral vessel permeability, thereby causing an epileptogenic effect on the brain parenchyma and leading to cerebral bleeding [134].

#### Blood-brain barrier disruption

The disruption of the blood brain barrier may suppose a leakage of pro-inflammatory agents and the accumulation of toxic agents (i.e. after cell death in ischaemic events or the presence of extracellular blood) can drive to the presence of seizures [135].

Toxic agents present in the extracellular matrix in the brain parenchyma can also activate intrinsic pathways and changes in the microvascular tone that could lead to headaches including migraine-like headaches [136].

#### Mass effect and rise of intracranial pressure

Any intracerebral hemorrhage may lead to headache through the increase in intracranial pressure, either due to the mass effect caused by the presence of bleeding or through secondary obstructive hydrocephalus, which can activate pain-sensitive structures and the trigeminovascular system due to meningeal stretching [137].

Blood stasis phenomenon, which would include cerebral venous thrombosis (CVT), dural arteriovenous fistula (DAVF), and arteriovenous malformations (AVM) can also lead to an increase in intracranial pressure. Blood stasis can lead to disruption of the blood-brain barrier, cerebral edema, ischemia, and intracerebral hemorrhage [138].

AVMs, especially large ones, can exert a mass effect on adjacent pain-sensitive structures, leading to the presence of headache and possible epileptogenic effects due to the compression of adjacent brain parenchyma [139].

There is not enough evidence to conclude that the excess pressure on the brain parenchyma can directly provoke seizures, however, hypothetically, these mechanisms especially if the onset is acute could result in such phenomena.

#### Mechanical activation of perivascular innervation

Mechanical activation of perivascular innervation could be responsible for headache found in ischemic stroke, mainly after revascularization procedures [140]. Mechanical stretching of the artery during endarterectomy, angioplasty, or stenting can activate the peripheral nerve supply, coming from the superior cervical ganglion, sphenopalatine and otic ganglia, and the trigeminal ganglion [136]. This extrinsic circulation plays a key role in keeping the blood vessel wall tone and releases vasodilator agents which have a protective effect against abrupt rising of blood pressure. Mechanic dilation of arteries during end arterial procedures could lead to the release of vasodilating substances such as neuropeptide Y, vasoactive intestinal peptide, acetylcholine, NO, nitric oxide synthase, CGRP, substance P, neurokinin A and pituitary adenylate-cyclase activating polypeptide. The release of those vasodilating substances may lead to headache and even migraine-like or trigeminal-autonomic-like headaches.

This mechanism could explain the presence of headache and triggered migraines in migraineurs in ischaemic stroke, endarterial procedures such as endarterectomy, angioplasty, angiography, and stenting and can also be one of the underlying mechanisms in cervical and vertebral dissections [141].

Additionally, vasculopathy is associated with inflammation of cranial arteries and may lead to the thickening of the vessel wall and activation of perivascular nociceptors, this mechanism can be seen in giant cell arteritis [132], PACNS, SACNS [133], and Sturge-Weber syndrome [129].

### The interictal burden in migraine and epilepsy

#### Cognitive and social aspects

Suffering from a chronic neurological disease might be responsible for academic failure and occupational underachievement, as well as it might affect cognitive function [142]. Recent literature studies suggested that migraine and epilepsy might be correlated with a decline in specific neuropsychological functions, encompassing attention, memory, and social cognition. Despite this, very few studies have explored cognitive abilities in subjects with both comorbid conditions. The patients suffering from one or both diseases are mostly of productive age and often have a low socioeconomic status. This influences not only the individual but also society, pointing out the importance of diagnosing and treating these diseases in early stages.

#### Cognitive dysfunctions in patients with migraine and epilepsy

There are hypotheses that migraineurs may have impaired cognitive ability. Prior research demonstrated an adverse influence of migraine, particularly migraine with aura, on executive functions like attention, verbal proficiency, psychomotor skills, and memory. Notably, the presence of migraine-related aura was correlated with cognitive deficits [143, 144]. Cognitive manifestations were prevalent during both the premonitory and headache stages of a migraine episode and may endure into the postdrome phase. The effectiveness of treatments aimed at mitigating acute attacks was not always successful at relieving cognitive symptoms. Additionally, cognitive impairment, especially in terms of executive function, played a role in the overall disability associated with migraine attacks [145]. Nevertheless, other studies did not reveal any discernible impact of migraines on cognitive abilities, and longitudinal studies failed to furnish substantiating proof for the gradual deterioration of cognitive functions in individuals with migraines over time [146]. In contrast, a study showed better cognition in migraineurs, especially with aura, in middle-aged patients [147]. Discrepancies observed among these studies could potentially stem from inadequate and different utilization of neuropsychological evaluations.

From the data in the literature emerged that most cognitive impairments observed in people with epilepsy were linked to the etiology of epilepsy and frequently arise from an interplay of diverse elements. Epileptic seizures could induce both structural and functional alterations in the brain, resulting in the presentation of cognitive and neuropsychological diseases. Furthermore, inadequate management and control of epileptic seizures could culminate in enduring cognitive dysfunction [148]. Epileptiform activity frequently exhibited correlations with deficits in memory function, cognitive delays, disturbances in communication and behavior, and compromised attention. Additionally, epileptiform activities had the potential to give rise to transitory or persistent deficits contingent upon various variables, including recurrent occurrences, intensity, the individual’s age, the modality of seizure prophylaxis, and its efficacy [149]. The enduring consequences of IED, accruing progressively, had the potential to induce substantial alterations in cognitive faculties, particularly impacting learning capabilities and memory, especially during evolutive age [149].

Only two studies in the literature examined cognitive abilities in subjects with comorbid headache and epilepsy. In the first study, it was noted that the presence of comorbidity in migraineous individuals with focal epilepsy did not correlate with any significant divergence in group performance concerning objective evaluations of cognitive impairment [150]. The second study demonstrated that the cognitive and neuropsychological characteristics of children suffering from migraine and Centro-Temporal spikes were influenced by factors beyond epilepsy itself, even though epilepsy itself appeared to play a role [122].

#### ADHD in patients with migraine and epilepsy

Regarding attention, several studies indicated a heightened occurrence of epilepsy and migraine among individuals diagnosed with attention deficit hyperactivity disorder (ADHD). Conversely, an elevated prevalence of ADHD was observed in clinical cohorts of children with somatic disorders [151]. Furthermore, the prescription trends for anti-migraine medications and medications targeting ADHD in adults suggested a potential association between migraine and ADHD. Although elucidating the reason for this connection is unfeasible, researchers have been speculating that this relationship might arise from shared underlying pathophysiological mechanisms [151].

The current theories concerning the pathophysiology and genetics of ADHD mainly focus on alterations of the dopaminergic system. Considerable substantiation was highlighted indicating the engagement of dopaminergic mechanisms in both migraine and mood disorders, thereby suggesting plausible shared etiological factors for these conditions [152].

It was hypothesized that the trigeminal nucleus caudalis generated migraines, whereas premonitory symptoms, encompassing sensations like hunger, fatigue, mood alterations, and sensory and visual distortions such as dizziness and aura, were largely attributed to hypothalamic dopamine. In individuals with migraine, a chronic insufficiency in presynaptic dopamine release was found, resulting in postsynaptic dopamine receptor hypersensitivity, so consequently, even normal to slightly elevated dopamine release levels can induce symptoms [151]. However, the pathogenesis of migraine is also influenced by environmental factors. Given that ADHD emerges before the age of seven, resulting in prolonged disruptions to social and academic functioning before the onset of migraine attacks, it is plausible that stress-related effects could contribute to the observed association between migraine and ADHD [151].

#### Social cognition in patients with migraine and epilepsy

Numerous investigations have underscored the prevalence of social cognition deficits in pediatric epilepsy, with heightened challenges being linked to an early onset of seizures. A recent comprehensive analysis demonstrated notable impairment in emotional recognition as well as the theory of mind in children and adolescents affected by epilepsy, a condition further associated with early-onset seizures and prolonged illness duration [153]. In contrast, only a limited number of studies have examined social cognition skills in the paediatric cohort afflicted by migraine [154]. Recent research indicated potential challenges in metacognition and theory of mind abilities among children with migraine when compared to their typically developing peers. However, the outcomes of the study remained inconclusive [155]. A single study in the literature explores social cognition in subjects with headaches and epilepsy [142]. This study suggested that children and adolescents with migraine or focal epilepsy showed difficulties in facial emotional recognition and theory of mind, compared to their peers. Both these difficulties seem to be potentially related to difficulties in executive functions [142].

#### Role of antiepileptic drugs

Cognitive decline may be a consequence of the treatment with antiseizure medications (ASMs) [148]. As with all pharmacological agents, patients taking ASM showed more pronounced side effects when combining more than one ASM or when taking higher doses of the drug. Interestingly, ASMs have been considered a risk factor for cognitive impairment [148].

One study unveiled an escalating probability of cognitive impairment in tandem with the augmented utilization of additional ASMs, with a more pronounced impact observed on executive functions. Consequently, the recommendation derived from the findings advocates for limiting the concomitant use of no more than two ASMs whenever feasible in therapeutic strategies [156]. Additionally, a research study indicated that older versions of ASMs, such as carbamazepine, valproate, and phenytoin, exerted a substantially greater deleterious influence on cognitive capacities compared to newer categories, barring the exception of topiramate [157]. Lamotrigine and levetiracetam were proved to be better options with less impact on cognitive processes, whereas topiramate had the most adverse effects on cognition [157]. It is worth noting that cognitive functions in individuals with epilepsy or headaches often exhibited inferior performance compared to healthy controls even before the initiation of ASM treatment. While limited in number, such studies underscored the diverse range of risks capable of influencing cognition even before the onset of diagnosis. Furthermore, the majority of investigations delving into the cognitive adverse effects of ASMs neglected to account for various other factors stemming from the inherent neurologic condition itself [158].

#### Role of psychopathology

Numerous community-based epidemiological investigations have indicated an elevated prevalence of depression, even within the population of individuals with epilepsy, thereby giving rise to adverse outcomes. A cross-sectional study of selected psychiatric symptoms among patients with new-onset focal epilepsy who have or do not have comorbid migraine highlighted that the co-occurrence of migraine and focal epilepsy is linked to the manifestation of depressive symptoms. Notably, no discernible contrast between the two groups was observed concerning symptoms of anxiety or depression [159, 160]. Manifestations of cognitive dysfunction, including deteriorated memory, psychomotor speed, attention, visual learning, and impaired executive functioning, were detected in individuals experiencing their initial episodes of depression. The most substantial impacts were evident in the domains of attention and executive function. Importantly, these cognitive symptoms persisted even during periods of remission when subjects were not undergoing mood disturbances, yet their cognitive performance remained distinct from that of the healthy control group [161].

In conclusion, there are strong indications that the comorbidity between epilepsy and migraine could exacerbate cognitive dysfunctions and deficits in executive functions, both due to pathogenetic, social, and therapeutic factors, especially if these disorders manifest during developmental ages. Therefore, further studies are required to assess potential heterogeneities in the underlying skills of cognitive abilities and the more conservatively oriented treatments for these functions. The identification of these factors may ensure an enhanced quality of life for affected individuals and mitigate the onset of psychopathological comorbidities.

#### Therapeutic similarities

The association between migraine and epilepsy treatment can be explained by shared pathophysiological pathways involving neuronal excitability, ion channel dysregulation, and neurotransmitter imbalances that contribute to both conditions [162]. Anti-migraine agents available today include tricyclic antidepressants, beta-blockers, calcium channel blockers, anti-CGRP drugs, and ASMs [163]. ASMs are efficacious in preventing migraine attacks, most probably due to their capability of blocking CSD, decreasing neuronal hyperexcitability, and potentially reducing the development of central sensitization [164]. They stabilize neuronal membranes and influence ion channels, which reduces the release of vasoactive neuropeptides, thereby diminishing neurogenic inflammation [164]. This chapter will focus on drugs proven effective in preventing migraine and epilepsy.

Topiramate (TPM) and valproic acid/sodium valproate (VPA) are the only two FDA-approved ASMs that have high-quality evidence supporting their use in preventing migraine [165]. TPM is indicated for various neurological and psychiatric conditions and is mainly used in adults and children with generalized or focal epilepsy [166]. Its miscellaneous mechanism of action involves inhibiting sodium and calcium channels [164]. Furthermore, the inhibition of neuroexcitatory glutamate-mediated circuits, crucial in CSD, along with the repression of CGRP secretion from the trigeminovascular system, probably justifies its anti-migraine properties [167]. The proof of the efficacy of TPM, at doses ranging from 100 to 200 mg/day, derives from numerous randomized controlled trials published in the last 20 years [165, 168].

VPA is a large-spectrum drug widely used in mood disorders and generalized and focal seizures, representing the mainstay of idiopathic generalized epilepsy therapy [169]. It has multiple cellular mechanisms of action including suppressing and blocking voltage-dependent sodium channels and increasing brain GABA concentrations [170]. Several trials demonstrated the efficacy of valproic acid at doses ranging from 500 to 1000 mg per day in migraine prevention [169]. However, caution is warranted in certain subpopulations. VPA is proven to have teratogenic effects and is associated with a significant increase in malformations, especially neural tube defects when used during the first trimester. It is also known to be excreted into breast milk, leading to sedation, irritability, weight loss, etc. in the breastfed infant. Therefore, it is recommended to use other ASMs in women of childbearing age [171, 172].

Other ASMs provided smaller and limited evidence suggesting their effectiveness in migraine prevention. Gabapentin (GBP) and pregabalin (PGB) are used to treat neuropathic pain and epilepsy [173]. They act by selectively inhibiting voltage-gated calcium channels containing the α2δ-1 subunit, with subsequent reduction of the release of excitatory neurotransmitters [174]. Although GBP and PGB are still used for migraine prevention based on clinical experience, a systematic review showed that they do not significantly reduce headache days frequency [173]. GBP, at a dose of 1800–2400 mg per day, only provided a minimal not-statistically significant benefit compared to active treatment [175].

Lamotrigine is a strong inhibitor of sodium voltage-sensitive channels and can suppress CSD, which helps prevent migraine aura symptoms [176]. However, it is not recommended for migraine-preventive therapy as clinical trials did not show a benefit in reducing headache attacks compared to placebo or TPM [177].

Zonisamide works similarly to TPM by affecting voltage-gated sodium channels and regulating GABA and glutamate neurotransmission [178]. It also targets calcium receptors located in the trigeminal ganglion and nucleus caudalis, which release CGRP [163]. Only a few small studies highlight its efficacy in migraine prevention [178].

Levetiracetam works by binding to synaptic vesicle proteins, particularly Synaptic vesicle glycoprotein 2 A, reducing neuronal hyperexcitability and blocking high-voltage-gated calcium channels [179]. Studies suggest that doses of 500–2000 mg per day can decrease the frequency, severity, and duration of migraine attacks, but more research is needed [180].

Carbamazepine (CBZ), a voltage-gated sodium channels inhibitor effective in focal epilepsies and trigeminal neuralgia, and oxcarbazepine, a derivate of CBZ [176], demonstrated uncertain or insufficient benefits in migraine prevention [181].

Perampanel, a noncompetitive AMPA antagonist, inhibits CGRP release from rat brainstem in vitro and it has been demonstrated as a promising therapy in 31 patients with migraine and epilepsy enrolled in an observational multicenter study [182].

In conclusion, exploring the pharmacodynamic mechanisms underlying the migraine prophylactic properties of ASMs, particularly their actions on CSD could help to understand the relationship between these two common neurological conditions.

## Conclusions

This review aimed to demonstrate the intricate relationship between migraine and epilepsy, which is underpinned by shedding light on the pathophysiological similarities that can be observed in phenomena like CSD, neurotransmitters, and genetic factors. The great number of clinical similarities and clinically overlapping syndromes is well explained by these shared pathophysiological mechanisms, however, distinguishing these diseases can be highly difficult in many cases, demonstrating the need for further research of the pathomechanisms and features of these diseases. This could enhance diagnosis and treatment and, as a result, improve the quality of life in many individuals affected by these disorders. To shed light on the quality of life, this review extensively addressed the interictal burden, showing that individuals suffering from one or both diseases are often highly affected in their social and cognitive abilities, resulting in significant costs and disadvantages not only for themselves but for society.

To put it in a nutshell, what becomes evident by exploring this relationship is that the intersection of migraine and epilepsy is not a coincidence but rather proof of the intricate web of the human brain’s functioning. Further research, a multidisciplinary approach, and highly educated clinicians using practical tools for diagnosis are warranted to improve the lives of those facing these conditions daily.

## Data Availability

No datasets were generated or analysed during the current study.

## Abbreviations

ADHD: Attention Deficit Hyperactivity Disorder
AMPA: α-amino-3-hydroxy-5-methyl-4-isoxazolepropionic acid
AMPAr: AMPA receptor
ASDH: Acute subdural haemorrhage
ASMs: Antiseizure medications
ATP1A2: ATPase Na+/K + Transporting Subunit Alpha 2
AVM: Arteriovenous malformation
Ca2+: Calcium
CAA: Cerebral amyloid angiopathy
CACNA1A: Calcium Voltage-Gated Channel Subunit Alpha1 A
CADASIL: Cerebral Autosomal Dominant Arteriopathy with Subcortical Infarcts and Leukoencephalopathy
CGRP: Calcitonin gene-related peptide
CSD: Cortical spreading depression
CT: Computer tomography
CVT: Cerebral venous thrombosis
DAVF: Dural arteriovenous fistula
EEG: Electroencephalography
FDA: Food and Drug Administration
FHM: Familial hemiplegic migraine
FHM1: Familial hemiplegic migraine type 1
FHM2: Familial hemiplegic migraine type 2
FHM3: Familial hemiplegic migraine type 3
FLAIR: Fluid-attenuated inversion recovery
fMRI: Functional magnetic resonance imaging
GABA: Gamma-aminobutyric acid
GCA: Giant cell arteritis
GWAS: Genome-wide association studies
HM: Hemiplegic migraine
ICHD-3: International Classification of Headache Disorders 3rd edition
IED: Interictal epileptiform discharges
IEH: Ictal Epileptic Headache
ILAE: International League Against Epilepsy
ICHD: International Classification of Headache Disorders
K+: Totassium
MRI: Magnetic resonance imaging
Na+: Sodium
PIH: Post-ictal headache
MELAS: Mitochondrial Encephalopathy, Lactic Acidosis and Stroke-like episodes
MMA: Moya-Moya angiopathy
NMDA: N-methyl-D-aspartate
NMDAr: NMDA receptor
NO: Nitric oxide
PACNS: Primary angiitis of the central nervous system
PRRT2: Proline - rich transmembrane protein 2
RCVS: Reversible cerebral vasoconstriction syndrome
RVCLSM: Retinal vasculopathy with cerebral leukoencephalopathy and systemic manifestations
SAH: Subarachnoid haemorrhage
SACNS: Secondary angiitis of the central nervous system
SCN1A: Sodium Voltage-Gated Channel Alpha Subunit 1
SHM: Sporadic hemiplegic migraine
SNP: Single nucleotide polymorphisms
TIA: Transient ischaemic attack
TRESK: TWIK-RElated Spinal cord K+
